# Tissue-specific aging—a new paradigm based on transcriptional insights from select model systems and humans

**DOI:** 10.3389/fgene.2026.1925373

**Published:** 2026-09-22

**Authors:** Stewart Frankel, Blanka Rogina

**Affiliations:** 1 Department of Biology, University of Hartford, West Hartford, CT, United States; 2 Department of Genetics and Genome Sciences, School of Medicine, University of Connecticut Health Center, Farmington, CT, United States; 3 School of Medicine, Institute for Systems Genomics, University of Connecticut Health Center, Farmington, CT, United States

**Keywords:** aging, cell atlas, cell type, gene expression, tissue-aging, transcriptome, tissue-specific, evolution

## Abstract

Aging is a complex process affected by genes and modified by environment. The forefront of aging research has moved from the level of organisms to the level of tissues and single cells, analyzed using bulk tissue and single-cell transcriptomes. Transcriptomics provides high resolution, comprehensive information per sample, and scalability to large numbers of samples. Datasets obtained from multiple tissues at multiple time points indicate that each tissue ages differently. The transcriptomic approach promises to greatly widen the identification of the candidate mechanisms and genes driving aging-related changes, which could then be tested in model organisms. Our focus here will be work in *C. elegans*, *Drosophila*, mice and humans, though considerable progress is being made in other organisms. Current research is translating molecular and physiological markers of tissue-specific aging into a wide range of diagnostics. An ultimate goal is to translate underlying mechanisms into therapies that will improve human health span.

## 1 Introduction

Aging has been viewed as an organism-wide decline in physiological function (Finch, 1990; Finch, 2009). In contrast, many diseases are frequently studied in an organ or tissue specific manner, such as heart disease (Cohn, 2018). The organism wide approach to aging and the organ specific approach to disease is now changing, since aging seems to be very organ/tissue specific, and particular organ-related diseases may have aging as one causative factor (Tian et al., 2023).

Aging at the organism level most frequently uses survival studies. The age of death for each individual in a population can be summarized by the mean and maximum life spans of the population, as well as age-specific mortality rates (Finch, 1990; Helfand and Rogina, 2003). Aging has also been studied by measuring changes in physiological functions with elapsed time (Reenan and Rogina, 2008; Rogina et al., 2007; Woods et al., 2014; Parashar and Rogina, 2009). However, most research on aging in model organisms and wild species has relied on longevity and other life history traits (Jones et al., 2014; Rogina et al., 2007; Frankel and Rogina, 2021; Promislow et al., 2022; Flatt, 2020). In the last 10 years new tools have allowed a more comprehensive study of aging at the level of organs, tissues and single cells. The results argue against a general decline of the body governed by mechanisms common to most tissues (with the exception of inflammation). Instead, each organ and tissue in an organism can age at a different rate, as defined by molecular profiles in mice, (Schaum et al., 2020); in rats (Shavlakadze et al., 2019), or a combination of molecular and physiological profiles in humans (Tian et al., 2023; Oh et al., 2023; Goeminne et al., 2025). Data supporting tissue specific aging may have been accumulating over decades but were embedded in studies on the progressive pathological changes associated with organ specific diseases. It has been postulated that some diseases may primarily affect the elderly due to a contribution of aging-related mechanisms to disease etiology (Ding et al., 2026). This review will highlight new aging research in *C. elegans*, *Drosophila*, the mouse, and humans, though important work is also being done in other model systems.

## 2 Aging at the organism and tissue level

Longevity is a complex trait affected by many genes and multiple environmental factors resulting in distinct trajectories when comparing different individuals, different populations, and different species (Helfand and Rogina, 2000; Frankel and Rogina, 2021). Different inbred, genetically homogeneous populations of laboratory mice and *Drosophila* show different longevity and mortality patterns. This heterogeneity was observed in both *ad libitum* controls and calorically restricted populations (Liao et al., 2010; Wilson et al., 2020), presumably due to the different alleles in each population as the environments are kept as uniform as possible. A recent study compared the transcriptome of 3–5-day old whole male and female flies to 3-week-old flies using the *Drosophila* Genetic Reference Panel (Tan et al., 2025), 200 inbred lines with different genetic backgrounds, to gain insight into how genetic variation affects age-related changes. The study found that age-, sex and genetic background had differing effects upon the transcriptome. When the genetic variance across all lines was partitioned, there was an age-related variance in expression for 64% the 10,806 expressed genes in females and 57% of the 14,261 expressed genes in males.

We postulate that a paradigm change is occurring in our understanding aging, driven by a shift in focus from the organism to the level of tissues and cells (Figure 1), a shift largely facilitated by the development of bulk RNA sequencing (bulk RNA), single cell RNA sequencing (scRNA-seq), and single nucleus RNA sequencing (snRNA-seq) (Kolodziejczyk et al., 2015; Lake et al., 2017; Bakken et al., 2018; Zeng and Dai, 2019; Zeng et al., 2016). Whole transcriptome sequencing is presently the best approach for capturing all of the variables at play in aging, due to the acquisition of large quantities of information, the ability to amplify this data from small samples, and the ability to scale up to many thousands of samples encompassing multiple tissue types, multiple time points during the lifespan, multiple individuals in the same inbred population, and multiple populations with different genetic backgrounds. Bulk RNA, scRNA-seq, and snRNA-seq have different strengths and drawbacks (Tzec-Interián et al., 2025).

**FIGURE 1:**
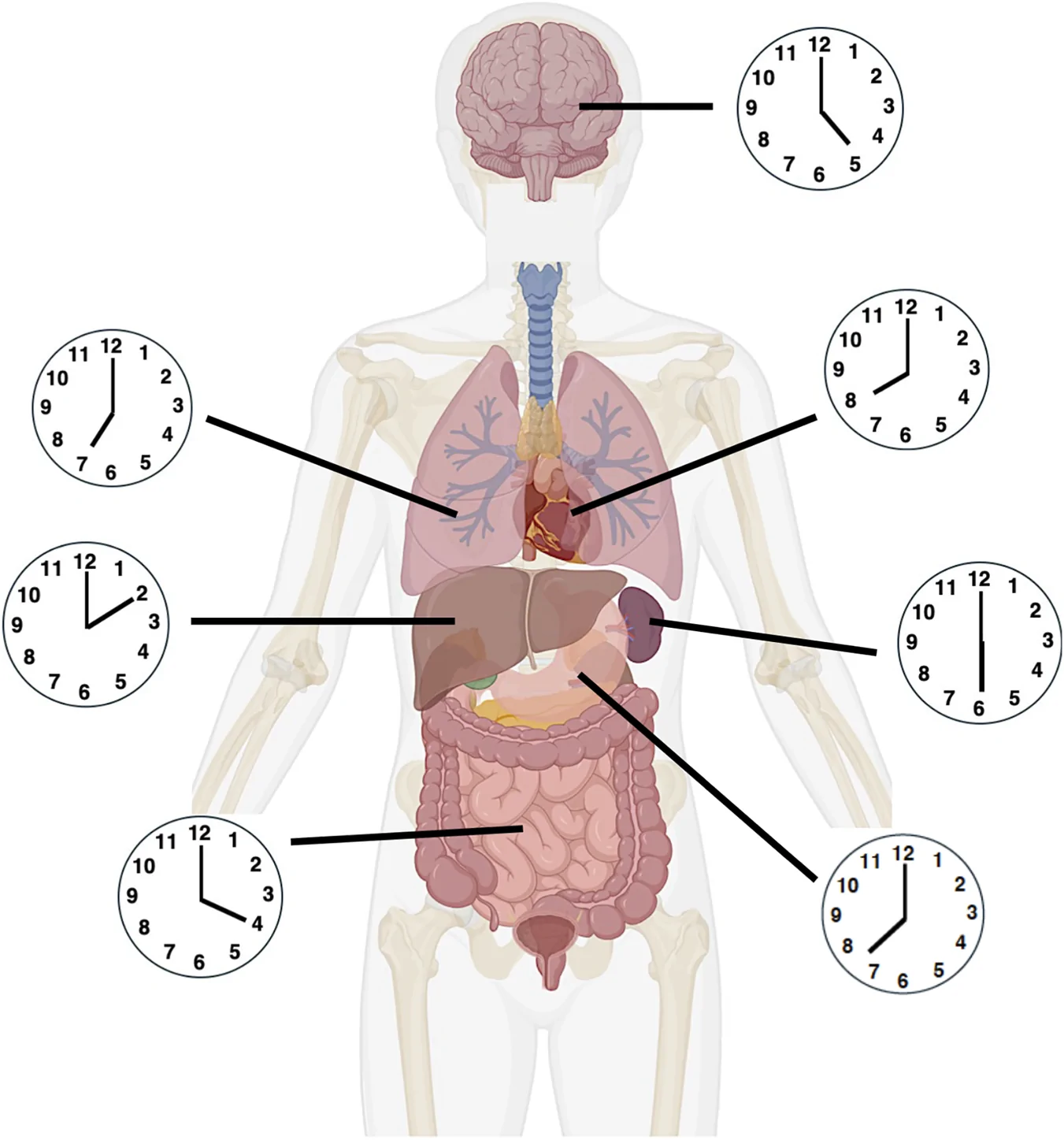
Tissue-specific aging. Multiomic datasets have been obtained from individual organs, tissues, or cell types, sampled at multiple times across the life span. Each organ ages at different rates and in qualitatively different ways. A similar situation is seen at the tissue and cellular levels. These multiomic changes have been used to develop organ, tissue, and cell type specific clocks that measure the rate of aging at these three different levels of the organism. The differing rates of aging in each organ are being shown by clockfaces connected to that organ. While the figure is a human, tissue specific aging has also been documented in model organisms. The times do not represent actual rates of aging. They only indicate that the rates of aging can differ in each tissue.

When studying aging at the tissue level, are the changes in transcription with advancing age autonomous to each cell, the result of changes in the cell types populating the tissue or organ, or both—questions that can be resolved using scRNA-seq (Kolodziejczyk et al., 2015; Zeng and Dai, 2019). The number of cells isolated and the number of sequencing runs are variables that affect the quality and significance levels of the transcriptomes. The sequences then need to be annotated or sorted into cell types. It is difficult to obtain intact cells from some tissues, requiring the isolation of cell nuclei and snRNA-seq. The nucleus has a smaller amount of RNA compared to the whole cell, reducing the signal and potentially reducing the richness of the dataset. In addition, the transcriptome will be skewed by the presence of RNAs only found in the nucleus (such as partially processed transcripts). snRNA-seq might therefore require extra sequencing runs and a more complex analysis process. However, the nuclei can be frozen, allowing easier sample collection when experimental design requires the isolation of hundreds of cells from multiple experimental samples. Several papers have compared the two techniques and found them comparable for some but not all applications (Lake et al., 2017; Bakken et al., 2018; Zeng et al., 2016).

Since whole organism aging can have several meanings, an acknowledged source of confusion (Mallapaty, 2024), we would like to propose the following description of tissue specific aging. One component is cell autonomous changes over the adult lifespan, where the changes involve molecules common to most cells in the organism. To fully capture the spectrum of cell or tissue specific changes, large scale molecular data will often be necessary. The other component would be cell non-autonomous changes, involving signaling, tissue architecture, and the mix of cell types present in a tissue with increasing age. Changes in the cell types present in tissues with increasing age has been intensively studied in mice (see Section 2.3) and humans (Sections 2.4 and 2.5). Spatial transcriptomics is providing information on changes in tissue architecture with age using molecular markers for particular cell types (Section 2.1).

### 2.1 Cell atlases, a new approach to defining cell and tissue types

Single cell and bulk RNA sequencing have been used to develop cell atlases, an inventory of all cell types present in the organism (*C. elegans* and *Drosophila*) or in the tissues sampled from the organism (mouse, human). Initial cell type annotations based upon the transcriptome can be further refined using proteomic, spatial, and other data. Atlases can be developed for any stage of life, but for aging studies the reference atlas will be one derived from young adults (Table 1). A statistical analysis based on principal component analysis (PCA) is used to cluster transcription profiles. Cell cluster data is visualized through dimensionality reduction methods such as UMAP or tSNE (Becht et al., 2018; Li et al., 2022). Different approaches are taken to the annotation process, depending on the model system.

**TABLE 1:** Reference cell atlases used for aging atlases in *Drosophila*, mouse and humans.

Cell atlases		
Drosophila*	Mouse**	Human***
Whole organism	Dissected tissues	Dissected tissues
5 days	3 months	Young ages vary by study
snRNA-seq	Bulk RNA-seq and scRNA-seq	scRNA-seq and snRNA‐seq

* (Li et al., 2022).

** (The Tabula Muris Consortium et al., 2018).

*** (The Tabula Sapiens Consortium et al., 2022).

Additional atlases published by The Human Cell Atlas consortium (Regev et al., 2017; Sikkema et al., 2023; Chen et al., 2024; Xu et al., 2025; He et al., 2024; Barnett et al., 2024; Ounadjela et al., 2024; Marečková et al., 2024; Bhat-Nakshatri et al., 2024; Yayon et al., 2024; Zhang et al., 2024; Elmentaite et al., 2021; Oliver et al., 2024; Kedlian et al., 2024).

For the *Drosophila* atlas, the assignment of marker genes and/or transcriptome patterns to particular cell types was done by a consortium of 40 laboratories and consensus voting (Li et al., 2022). 250 major cell types were identified, though most had subtypes based upon the PCA and tSNE analyses. The sequencing used for the whole body atlas (Li et al., 2022) and a brain-only atlas (Davie et al., 2018) had a high level of genome coverage, since over 12,000 protein-coding genes were detected in each study. The number of major cell types may increase when deeper sequencing reads are obtained. For the mouse cell atlas, 20 tissues were isolated and dissociated into cells (The Tabula Muris Consortium et al., 2018). Cell cluster identification used a library of known marker genes, as well as other genes known to be differentially expressed. Cell type analysis was done independently for the sequences obtained from three different cell isolation procedures and compared. The mouse cell atlas consortium (The Tabula Muris Consortium et al., 2018) contains a large number of research groups and was able to draw upon their expertise for cell annotation. For *C. elegans* there were 211 cell clusters after the UMAP stage (Roux et al., 2023). Wormbase contained 5,375 unique genes linked to 2,438 anatomical terms, based upon GFP-labeled genes or *in situ* expression data. Further analysis of these anatomical markers linked each of the 211 clusters to a particular cell type; for some clusters there was one cell per worm.

A cell’s function includes its location in a tissue and its interactions with other cells. While the transcriptome can be a unique identifier of cell type, it is just the first step in describing a cell’s phenotype (Zeng, 2022). Spatial transcriptomics allows expression data to be mapped across thin tissue slices. One approach adds a lattice to the tissue slice, where each well has the sequencing reagents (Rao et al., 2021; Gulati et al., 2025). A different approach uses a modified FISH (fluorescence *in situ* hybridization) technique where gene-specific fluorescent probes are hybridized, quantitatively visualized, and then cleared, a process repeated multiple times (Rao et al., 2021; Gulati et al., 2025). This second approach has been used for aging studies. Hundreds of cell type marker genes were used to map the aging of all tissues in *Drosophila* (Janssens et al., 2025) and the brain of mice (Sun et al., 2025).

### 2.2 Aging cell atlases in flies

Since transcription patterns change with age, an aging cell atlas needs to track cell types against the background of a dynamic transcriptome. For instance, approximately 80% of 163 cell types in young adult *Drosophila* show an age-associated decrease in the number of expressed genes (Lu et al., 2023). In addition, the level of expression of marker genes for most cell types decreased significantly with age, though most did not decrease to zero. For some cells it was useful to have marker genes for the young ages and different markers for the old ages, with a clear transition at intermediate ages. The Aging Fly Cell Atlas was performed using snRNA-seq, since intact cells could not be obtained for various tissues (Lu et al., 2023). The aging fly atlas detected most of the cell types described in the reference fly cell atlas, including 91 cell types in the head and 72 in the body (Lu et al., 2023). Flies were sampled at 5 days (young flies), 30, 50, and 70 days (see Table 2). The following trends were observed. (1) The identity of the genes differentially expressed with age (differentially expressed genes, DEGs) and their GO designations were specific for each cell type (112 cell types had sufficient data for this analysis). (2) The fat body cell type had the largest number of DEGs, about 4,000. *Drosophila* fat body cells have the properties of both mammalian fat cells and hepatocytes, regulating metabolism throughout the organism. The number of DEGs is much lower for most cell types, with only 18 having 1,000 or more, and the rest roughly averaging 100. (3) In addition to the decrease in the number of expressed genes, all genes that continued to be expressed had decreased levels of transcription with age. (4) Genes related to protein translation decreased in all cell types, the only conserved GO feature common to all cell types. (5) An aging clock, or a clock of biological age, was modeled using transcriptome changes. 80% of the DEGs used by the aging clock were cell type specific DEGs.

**TABLE 2:** Aging cell atlases in Drosophila, mouse and humans.

	Drosophila#	Mouse##	Mouse###	Human
	snRNA-Seq	Bulk	scRNA-Seq	—
	scRNA-Seq	RNA-Seq mouse ages in months	—	equivalent age in years
Young adult, reference	5 days	3	3	20
Young adult		6		30
Young adult		9		36
Middle ages		12		43*
		15		50
	30 days	18	18	56
Older ages		21	21	63*
	50 days	24	24	69
		27		75
	70 days		30	81

* Multiomics shows peaks of aging-related changes at these ages (part IV, of Table 3).

#(Lu et al., 2023; Davie et al., 2018).

##(Schaum et al., 2020).

###(The Tabula Muris Consortium et al., 2018).

Abbreviations: scRNA-seq, Single-cell RNA, sequencing; snRNA-seq, single-nucleus RNA, sequencing; bulk RNA-seq, bulk RNA, sequencing.

Two studies in flies illustrate the strengths and drawbacks of scRNA-seq versus bulk RNA seq. One study used scRNA-seq of fly brain cells dissected from males and females of two different strains, using eight time points across the life span (Davie et al., 2018). The other study used bulk RNA sequencing of the head of male flies in one strain at 15 time points (Hanson and Macdonald, 2025). Both studies found a global decrease in transcription with age. Bulk RNA of the whole heads allowed easier collection of samples but lacked cell-specific transcriptomic information. scRNA-seg requires brain dissection, decreasing the yield of viable cells and limiting the number of organisms that can be sampled. In the scRNA-seq study, markers indicated that neuron cell identities were very stable with age. In the aging atlas study neurons also showed the fewest changes, while non-neuronal cells had higher levels of change. The study using bulk RNA sequencing of the head detected 6,142 age-related DEGs, 50% of the total genes detected. The authors attribute this to the higher number of time points, since fewer DEGs are obtained if they restricted their comparisons to just the youngest and oldest flies. Two additional factors may contribute to the higher number of DEGs: scRNA-seq may not include rare cells and whole heads contain several complex tissues in addition to neurons: compound eyes, antennae, proboscis, and cuticle. This bulk RNA study confirmed that stress response and immunity-related genes had increased expression with age; these results are consistent with previous bulk and scRNA-seq studies on *Drosophila* (Frankel and Rogina, 2021). However, the major contribution from using 15 time points was a clearer view of DEG dynamics over time. There were 28 distinct trajectories, five showed continuous decreases with age, five showed continuous increases with age, and some showed increases or decreases limited to early or late ages. A few trajectories showed peaks at intermediate ages or inflection points at mid-life, details that might be lost when fewer ages were sampled. Interestingly, similar temporal patterns of gene expression during aging were observed in the antenna of adult *Drosophila* measured by using enhancer trap and reporter gene constructs that marked 49 different genes (Helfand et al., 1995).

### 2.3 Aging cell atlases in the mouse

One study from the single-cell mouse atlas consortium (The Tabula Muris Consortium et al., 2020) analyzed changes in the cell type composition of aging tissues. As was seen in the fly studies, there was a global decrease in transcription with age and a decrease in the number of expressed genes (Zhang et al., 2021). Another consortium-related study did bulk sequencing of 17 organs (Schaum et al., 2020) at 10 ages in males and eight ages in females to determine DEG patterns, see Table 2. The DEGs that first became apparent at 6 or 9 months of age (equivalent to 30 or 36 year old humans) remained qualitatively the same in older animals, but with steadily increasing quantitative changes as the animals aged. In addition, transcription factor regulatory networks were largely unique to each tissue. The expression of each DEG was plotted as a function of age, using data from each organ separately and all organs pooled together. There were eight patterns or trajectories, matching some of the 28 trajectories in the *Drosophila* brain bulk RNA study. Genes from every organ followed these patterns but showed fine-grained organ-specific differences. scRNA seq was then used to determine whether DEGs in a particular organ could be due to an increased abundance of particular cell types with age. The increased expression of genes associated with inflammation in fat, kidney, heart, liver, muscle, bone marrow, and spleen tissues correlated with an increase in B cells. A separate publication focused on scRNA-seq of the mouse brain at young (2–3 months) and old (21–23 months) ages (Ximerakis et al., 2019). Twenty-five cell types were identified based on unique molecular identifiers. The cell types were preserved with age, though some of their identifiers had large changes in expression with age. 7.6% of the genes showed age-related changes, most commonly affecting cellular respiration, protein synthesis (ribosomal genes), and inflammatory responses.

Two more recent papers provide additional data on changes in the numbers of murine cell types with age (Sziraki et al., 2023; Zhang et al., 2025). The first paper looked at the brain, the second non-neural tissues. These two studies performed additional snRNA-seq on 14 mouse tissues at three ages, used different techniques for clustering sequence data, and the brain study included chromatin accessibility data for all sequenced cells. The result was a much higher signal for different cell types in each tissue, including rare cell types. 270 main cell types were identified in 14 organs, though there was a total of greater than 3,000 different transcriptomic/chromatin states. 23 of the 239 cell types analyzed in non-neural tissues showed significant changes in cell number with age. Mice in “advanced adulthood” (12–23 months, equivalent to 40–70 years of human age) showed a large increase in the abundance of multiple types of immune cells. While the overall number of immune cells increased with age, only a limited subset B and T cells were involved; other types of immune cells decreased. This is consistent with many studies at the organism level that show increased inflammatory immune responses at later ages, and in general a more dysregulated immune system (Frankel and Rogina, 2021). The same research group has recently created an aging atlas using chromatin accessibility, a novel approach (Lu et al., 2026). Single-nucleus ATAC-seq was performed on 21 mouse tissues at three ages. This technique isolates and clones transposase accessible regions of chromosomes. The ATAC-seq data was then clustered using sequences associated with cell type markers. Annotations were validated by comparisons to snRNA-seq clusters. Transcription factor motifs and cis-regulatory elements relevant to each cell type were identified and analyzed for age-related changes. They found that 91% of the epigenetic changes associated with aging were cell type specific. The observed chromatin changes are a first step towards determining the mechanisms causing aging-related shifts in gene expression.

Several of the studies on aging tissues in rodents focus on gene expression changes common to multiple tissues, though tissue specific changes were also mentioned. The authors of the study in rats (Shavlakadze et al., 2019) mentioned that many age-related gene expression changes were tissue specific. One of the mouse aging studies (Zhang et at., 2021) examined tissue specificity using six general categories: endothelial, epithelial, immune, parenchymal, stem cell, and stromal cell tissues. Four of the six categories had category-specific DEGs (60–80). It is difficult making cross species comparisons of tissue specific age-related changes. For example, in one mouse study the gene expression changes for each tissue were presented as self-organizing maps of Spearman’s coefficients (Schaum et al., 2020). These maps showed that gonadal, mesenteric, and subcutaneous fat had the greatest number of age-related changes. However, the same paper shows that fat tissues also have among the highest age-related increases in immune cells. Fat cells in the fly showed the highest number of DEGs, but an analogous analysis of immune cells and immune function was not performed.

In order to have a new model organism more closely related to humans, a cell atlas of the smallest lemur species has been published; this species has an 8 months generation time (The Tabula Microcebus Consortium et al., 2025). scRNA-seq of 27 organs that yielded 750 cell types.

### 2.4 Human cell atlases

The formidable task of assembling a human cell atlas has been undertaken by two separate consortiums (Human Cell Atlas consortium or HCA, Regev et al., 2017; The Tabula Sapiens Consortium et al., 2022), each using different strategies. Another draft atlas was compiled by a group not affiliated with either consortium, using 15 tissues from one adult donor and an average of 5,000 cells per tissue (He et al., 2020). The Tabula Sapiens consortium atlas aimed for at least 10,000 cells per tissue, while the HCA consortium has often had much higher numbers of cells per tissue. A third consortium (GTEx) has been gathering transcriptome data from 49 human tissues gathered postmortem, with the goal of systematically mapping genetic traits at the molecular level, ultimately translating quantitative human genetics into a bank of molecular data that can be analyzed and queried (Lonsdale et al., 2013; The GTEx Consortium et al., 2020). The transcriptome and epigenetic data gathered by the GTEx consortium could potentially be used to enhance the databases of the atlas consortiums.

The Tabula Sapiens consortium (The Tabula Sapiens Consortium et al., 2022) developed a single cell transcriptome atlas for 24 adult tissues, donors ages ranged from 22 to 74. Two methods of cell type annotation were compared to allow the detection and correction of potential errors: manual annotation by a large panel of experts and automated annotation using a method developed by the consortium. 475 cell types were identified. Most of the tissues from two donors were also prepared for histological examination by a team of pathologists, which classified cells as either epithelial, endothelial, immune, or stromal; there was a partial correlation of transcriptomics and histology.

The HCA consortium (Regev et al., 2017; Rood et al., 2025) has focused on studies of particular tissues—normal, diseased, and developing. Starting with an outline of the full project (Regev et al., 2017), the HCA consortium has published detailed atlases of the lung (Sikkema et al., 2023), brain (Chen et al., 2024), endoderm-derived organoids (Xu et al., 2025), neural organoids (He et al., 2024), vascular cells (Barnett et al., 2024), first trimester placenta (Ounadjela et al., 2024), endometrium (Marečková et al., 2024), female breast (Bhat-Nakshatri et al., 2024), the development of the thymus (Yayon et al., 2024), embryonic limb (Zhang et al., 2024), the adult gastrointestinal tract (Elmentaite et al., 2021), aging skeletal muscle (Kedlian et al., 2024; Lai et al., 2024), aging skin (Zou et al., 2021), aging ovaries (Zhou et al., 2024), aging peripheral blood mononuclear cells (Terekhova et al., 2023), as well as other tissues. The muscle, skin, and blood studies are examples of using readily biopsied tissue to study the aging of that tissue. The other cited studies used biopsies, donor tissue, organoids, or in the case of brain study, data downloaded from 70 human brain studies and 103 mouse brain studies.

### 2.5 Aging human tissue atlases

Tissue specific aging in humans will be illustrated with the results from two skeletal muscle studies. One used skeletal muscle biopsied from the limb (Lai et al., 2024), the other intercostal muscles (Kedlian et al., 2024); both used scRNA-seq and snRNA-seq. The limb study also generated snATAC-seq data; their tissue was donated by 12 young (15–46 years) and 19 older adults (74–99 years) with different fitness and frailty levels. Kedlian et al. (2024) used tissue from eight young (20–40 years) and nine older donors (60–75 years). Both groups found an almost total loss of Type IIX fast twitch muscle fibers in the older cohort (the two studies had different findings for the other type of fast twitch fiber) and identified some slow twitch fibers that had developed a fast twitch molecular profile (a potential compensatory mechanism). Intercostal muscle fibers from the older donors had decreased gene expression and smaller diameters. The limb muscles showed a decrease in quiescent stem cells and increased number of activated stem cells with inflammatory characteristics, while the intercostal muscles showed a stable population of quiescent cells and decreased number of activated cells. Each stem cell change was viewed as contributing to the known decrease in muscle fiber regeneration with age. Both groups found increased proinflammatory immune cells in both the muscle and vasculature compartments of the tissue. The limb muscle study correlated the transcriptome and ATAC-seq data, identifying putative gene regulatory networks and relevant transcription factors. This data set was viewed as a foundation for linking chromatin changes to genetic variants known to affect muscle function.

## 3 Whole organism and tissue specific aging clocks

Molecular and clinical data have been used to develop aging clocks, which predict either chronological or biological age (Horvath and Raj, 2018; Rogina, 2021; Johnson and Shokhirev, 2024; Min et al., 2024; Srour et al., 2025). The latter is defined in terms of how much change occurs as the organism ages. An increased age-associated change in the phenotype of one organism or tissue compared to another is considered a case of accelerated biological aging. Most aging clocks have been focused on humans. Whole organism clocks have been constructed using various types of inputs such as DNA methylation, transcriptome, or blood proteome data, followed by machine training to “tune” the clock. One study used data from the ATAC-seq (Morandini et al., 2024). Tissue specific aging clocks in humans have used either transcriptome data (Shokhirev and Johnson, 2021), the plasma proteome (Oh et al., 2023; Goeminne et al., 2025; Ding et al., 2026), clinically-relevant diagnostic tests that are organ-specific and related to organ function (Nie et al., 2022), or complex data sets that include plasma proteins, plasma metabolites, routinely measured physiological tests (such as blood pressure), and brain scans (Tian et al., 2023). Another study examined age-related changes in multiple massive datasets; all had changes that peaked at approximately 44 and 60 years of age (108 individuals were studied) (Shen et al., 2024). See Table 3 for descriptions of several of these studies; the chronology indicates how rapidly this field is advancing.

**TABLE 3:** Measuring organ-specific aging in humans and correlation with organ-specific diseases.

I. Leveraging plasma protein levels for prediction of organ age and organ disease.
Genes specific to 11 organs were identified using the GTEx transcriptome databank, where 4-fold higher expression in one organ was used as the criteria of specificity. Out of 5000 detectable plasma proteins, 856 were the products of the organ-specific genes. Using biobank sample donors of multiple ages and the levels of organ-specific proteins in each sample, regression models were trained to determine chronological age. The models were tested on other biobanks for age accuracy. Plasma proteins not specific to an organ were used to train models for whole organism age. Each organ aged at a different rate from other organs. The ages of organs in individual samples were then compared to the population mean, to determine age gaps. Large positive gaps meant extreme aging for the organ in that individual. The physiological significance of such gaps was tested using 9 age-related disease states. For several organs there was a strong correlation between the age gap and either the presence of disease or the development of disease in the same individual at a later age (Oh et al., 2023).
II. Leveraging plasma protein levels for prediction of mortality, or organism age, or organ-specific disease.
Data for 3000 plasma proteins was obtained from the UK Biobank for 45,000 participants. The participant pool was divided into fifths, and each fifth was trained and then tested against the rest of the pool. One model was trained to predict for chronological age and another to predict mortality. Both organism-wide models predicted a higher incidence of age-related diseases with age. Each plasma protein was correlated with either age or mortality using regression analysis. Organ-specific proteins, defined as in Oh et al. (2023), were the input for model training. Organ biological ages, as derived by the organ model, showed significant correlations of varying strengths with each other and chronological age. Increased organ specific aging was strongly correlated with increased incidence for organ specific diseases. Environmental factors affected modeled organ aging (Goeminne et al. 2025).
III. Leveraging physiological data to predict organ specific diseases.
This study was focused upon common and widely used diagnostics to model aging of individual organs, and model how one organ’s aging can affect other organs. Data used: blood plasma biomarkers for the immune, renal and hepatic systems; metabolites in the blood; routinely measured physiological tests for cardiac, pulmonary, muscular function; brain scans. These were used as input for machine learning/training to determine age gaps (accelerated aging compared to the population average). A wide range of genetic, environmental and life-style factors affected age gaps. Age gaps for some (but not all) organs predicted elevated risk of disease for that organ. Interaction networks were mapped, where one organ’s age gaps predict elevated risk for disease in certain other organs. The tools created by this study have the potential to significantly enhance current diagnostics and the targeting of interventions before the onset of disease (Tian et al 2023).
IV. Using multiomic data as input for the analysis of human aging.
The following data were used as input for machine learning algorithms and machine-based statistical analysis. Bulk RNA-seq of peripheral mononuclear blood cell; plasma proteomics; plasma metabolome; comprehensive panels of clinical blood tests; plasma cytokine levels; stool, skin, oral, and nasal microbiomes. Aggregated analysis shows two peaks of “extended molecular changes” around 40 and 60 years (Shen et al 2024).
V. Leveraging plasma protein levels for prediction of cell type age, and the utility of predicting particular diseases and mortality based upon the ages of particular cells.
This study built upon the work in Oh et al. (2023) after the formation of the Global Neurodegeneration Proteomics Consortium (GNPC) in 2023. Cell-type specific proteins were identified in the human transcriptome databank, defined as the two times higher expression in one cell type. 1202 proteins were cell type specific using a platform that could detect 7289 proteins (a different platform was also used). Each cell type had proteins that showed distinct age-related expression patterns. Modeling programs were trained on a healthy biobank cohort to predict cell type age based upon the plasma protein profile, similar to what was described for organ-specific age in the earlier study. This resulted in models for 43 cell types. The models were able to detect increased and decreased aging rates in individuals compared to the cohort average, called gaps. Correlating positive gaps (increased cell aging) to neurological and muscular diseases was a priority for the GNPC. A positive gap in skeletal myocytes highly correlated with ALS. Aging of inhibitory but not excitatory neurons was correlated with AD. Many other correlations were identified. These gaps had predictive power. Asymptomatic individuals with highly positive gaps for astrocytes had a 12.6-fold higher rate of AD at later ages. Such gaps also correlated with environmental and genetic risk factors, such as APOE genotype. Gaps were also able to predict increased chances for various cancers, type 2 diabetes, and COPD, heart failure, stroke, and all cause mortality (Ding et al. 2026).

Aging clocks raise the issue of correlation versus causation. The first highly accurate organism clocks were based on DNA methylation (Horvath and Raj, 2018). A recent review has argued that methylation clocks, and maybe transcriptomic clocks, are measuring stochastic change (Meyer et al., 2025). The multiomic human clocks based upon the blood proteome and physiological data might be measuring residues left by trails of causation, though further research will be needed before any link to mechanisms of aging and disease etiology can be made.

## 4 Tissue-specific aging from an evolutionary perspective

Age-related changes specific to each type of tissue, which have been used to construct tissue-specific aging clocks in humans, are consistent with evolutionary theory. Aging has been proposed to be a byproduct of the evolution of the life history of a species (reviewed in Frankel and Rogina, 2021). For most but not all species, this means there will be a post-reproductive period of the life span subject to little selection pressure for fitness. Mutation accumulation and antagonistic pleiotropy have been proposed as two outcomes from the loss of fitness selection later in life (Frankel and Rogina, 2021). If a gene is expressed throughout adult life, there would be selection against non-neutral mutations in the coding region, but mutations could accumulate in regulatory sequences that affect late life expression. Increased or decreased late life expression is a common pattern for the DEGs identified by bulk and scRNA-seq in *Drosophila* and the mouse. It is possible that a mutation could cause ectopic expression at late ages of a gene that would normally not be expressed in a particular cell type. An evolutionary perspective on aging would predict a different aging process in each tissue due to the greater probability of mutations affecting gene regulation. Work on scRNA-seq in *C. elegans* (Roux et al., 2023) and other organisms add another mechanism that can drive the changes in gene expression observed in late life: resilience in response to stress and damage. Proposed mechanisms for resilience are present throughout adult life but would become greatly upregulated later in life. In *C. elegans* genes involved in DNA repair are upregulated, ribosomal protein genes are up or downregulated depending upon the tissue, mitochondrial function is downregulated, and protein homeostasis shows complex regulation (ER-UPR down, proteostasis down, chaperones up). The resilience profile appears to vary between cell types. Supporting this mechanism are enhanced resilience pathways in long lived species such as the naked mole rat (Zhao et al., 2018; Sahm et al., 2018; Chen et al., 2025).

If lifespan is used as a proxy for aging, its genetic component or heritability can be calculated from lifespan data. A recent paper strengthened the case for applying the evolutionary model, where germline mutations drive aging, to humans by showing 55% heritability (Shenhar et al., 2026). The environmental component of lifespan is extrinsic mortality (accidents, infections, unhealthy aspects of life-style that can cause pathology, and predation in wild animals). Intrinsic mortality would be linked to changes in body functions that lead to death. The heritability of lifespan was mathematically modeled using data from monozygotic and dizygotic twins, thereby maximizing the genetic component. The twins were born between 1900 and 1935, a period during which extrinsic mortality was estimated to decrease 3-fold, allowing it to be mathematically subtracted from the survival models. An intrinsic heritability of lifespan of 55% fits the heritability obtained from outbred mouse strains (Klebanov et al., 2000). The mutation accumulation theory postulates that most mutations affecting late life will be negative. While it is worthwhile to search for protective mutations leading to late life health, identifying mutations with negative effects may help diagnose late life outcomes in humans.

## 5 Looking ahead

Among the various goals for studying aging, perhaps the most prominent is to postpone physiological decline and maximize human health span so that the elderly can function effectively without prolonged illness. Evolutionary theory would argue for a focus on tissue specific aging in humans. It is probable that germline mutations contributing to human aging will be species specific, as that seems to be the general cross-species pattern and human life history has undergone dramatic changes compared to other primate species. Through the work of the GTEx consortium mutations can be identified in gene regulatory regions that act during the latter half of the life span (Lonsdale et al., 2013; The GTEx Consortium et al., 2020). These could then be linked to aging in particular tissues. The eventual goal would be to scan an individual’s genome sequence for such mutations, guiding diagnostics and potential therapeutic interventions.

The transcriptome data indicates there is increased inflammation and other marked changes as the immune system ages. There is also an increased infiltration of immune cells in many tissues. One of the studies on the aging of human skeletal muscle illustrates two additional aging mechanisms, each with tissue specific and non-specific mechanisms: stem cell dysfunction and the accumulation of senescent cells (Kedlian et al., 2024;^,^ López-Otín et al., 2023). They used a primary myoblast culture model to confirm the following findings from cells isolated from tissue. Sixteen genes important for muscle cell differentiation were used as markers for active and quiescent stem cells. There was decreased muscle stem cell activation with age. In addition, cultured stem cells from older patients showed signs of senescence. Primary cell senescence is a cell autonomous change, and mainly affects fibroblasts, endothelial cells, and immune cells (López-Otín et al., 2023; Suryadevara et al., 2024). All tissues have endothelial cells, and most aging tissues have immune cells. Secondary senescence is cell non-autonomous, the result of signals released from senescent cells (known as the senescence-associated secretory phenotype or SASP) into the tissue microenvironment (Gorgoulis et al., 2019; López-Otín et al., 2023). Given the evidence that certain types of immune cells increase in many tissues with age, secondary senescence may have both a tissue-specific trajectory, due to the type of immune cells present and the tissue microenvironment, and features shared across tissues, such as particular signaling molecules. The SenNet Working Group is providing guidelines for the detection and characterization of senescent cells in a wide range of human and mouse tissues using particular markers (Suryadevara et al., 2024; Barros et al., 2021). This will greatly enrich the understanding of tissue specific aging in humans. From a therapeutic viewpoint, such data can be used as a guide for the development of senolytic drugs (Zhang et al., 2023; Zhang et al., 2026).

Increased inflammation and a progressively more dysregulated immune system is well documented during aging in humans (Singh et al., 2024; Andonian et al., 2025) and mice (Krishnarajah et al., 2022). Since inflammation can be a normal part of the innate immune response and occur in response to stress, teasing apart a more frequent occurrence of appropriate inflammation in the elderly versus chronic, inappropriate and harmful inflammation can be difficult. Tissue specific aging studies utilizing single cell transcriptomes and cell atlases have transformed the study of inflammation and immune system changes with age, forming a foundation for the rational design of therapies for the harmful types of change. Another area of aging research concerns a change in the state of cells called mesenchymal drift (MD) (Lu et al., 2025). This change is based upon a gene signature that is shown to occur in a wide variety of aging tissues and is detected in various diseases associated with advanced age or organ failure. This gene signature occurs in normal human fibroblasts and mice at advanced ages and can be reduced by treatment or induction respectively of Yamanaka factors, at a dose too low to produce pluripotency but sufficient to change the state of the cells. This area of research also has great therapeutic promise.

Hovering over these projected applications is the theory that age-related changes are a significant mechanism driving particular diseases, maybe the major factor when both autonomous and non-autonomous effects are considered. Altering the rate of aging-related changes in specific human tissues will require extensive information about the underlying mechanisms. Aging-related changes in chromatin structure are one set of candidate mechanisms that can be targeted therapeutically (Lu et al., 2026). There are already a range of drugs that alter chromatin structure, making this a promising area for future drug development.

### 5.1 Conclusion

A paradigm shift from whole organism aging to tissue specific aging (Figure 1) has been greatly accelerated by new data that can track detailed molecular changes at the single cell level. Each tissue follows a different aging trajectory, as can be seen most clearly using high resolution transcriptome techniques. Diagnostic tools that can track the aging rates of each human tissue hold great promise for clinical interventions at much earlier stages in disease progression than was ever considered possible. As more evidence is obtained linking tissue specific aging to diseases of the elderly, the therapeutic modulation of the underlying aging mechanisms would significantly widen the ability to improve human healthspan.
