# Correction: Tissue-specific aging—a new paradigm based on transcriptional insights from select model systems and humans

**DOI:** 10.3389/fgene.2026.2008131

**Published:** 2026-10-08

**Authors:** Stewart Frankel, Blanka Rogina

**Affiliations:** 1 Department of Biology, University of Hartford, West Hartford, CT, United States; 2 Department of Genetics and Genome Sciences, School of Medicine, University of Connecticut Health Center, Farmington, CT, United States; 3 School of Medicine, Institute for Systems Genomics, University of Connecticut Health Center, Farmington, CT, United States

**Keywords:** aging, cell atlas, cell type, gene expression, tissue-aging, transcriptome, tissue-specific, evolution

In the following sentence, Table 1 was cited instead of Table 2.

“Flies were sampled at 5 days (young flies), 30, 50, and 70 days (see Table 1).”

A correction has been made to section 2.2, paragraph 1, on page 4

“Flies were sampled at 5 days (young flies), 30, 50, and 70 days (see Table 2).”

Here is the complete paragraph with the correction.

Since transcription patterns change with age, an aging cell atlas needs to track cell types against the background of a dynamic transcriptome. For instance, approximately 80% of 163 cell types in young adult *Drosophila* show an age-associated decrease in the number of expressed genes (Lu et al., 2023). In addition, the level of expression of marker genes for most cell types decreased significantly with age, though most did not decrease to zero. For some cells it was useful to have marker genes for the young ages and different markers for the old ages, with a clear transition at intermediate ages. The Aging Fly Cell Atlas was performed using snRNA-seq, since intact cells could not be obtained for various tissues (Lu et al., 2023). The aging fly atlas detected most of the cell types described in the reference fly cell atlas, including 91 cell types in the head and 72 in the body (Lu et al., 2023). Flies were sampled at 5 days (young flies), 30, 50, and 70 days (see Table 2). The following trends were observed. (1) The identity of the genes differentially expressed with age (differentially expressed genes, DEGs) and their GO designations were specific for each cell type (112 cell types had sufficient data for this analysis). (2) The fat body cell type had the largest number of DEGs, about 4,000. *Drosophila* fat body cells have the properties of both mammalian fat cells and hepatocytes, regulating metabolism throughout the organism. The number of DEGs is much lower for most cell types, with only 18 having 1,000 or more, and the rest roughly averaging 100. (3) In addition to the decrease in the number of expressed genes, all genes that continued to be expressed had decreased levels of transcription with age. (4) Genes related to protein translation decreased in all cell types, the only conserved GO feature common to all cell types. (5) An aging clock, or a clock of biological age, was modeled using transcriptome changes. 80% of the DEGs used by the aging clock were cell type specific DEGs.

The original version of this article has been updated.

